# The Deubiquitinating Enzyme Inhibitor PR-619 is a Potent DNA Topoisomerase II Poison[Fn FN3]

**DOI:** 10.1124/mol.119.117390

**Published:** 2019-11

**Authors:** Ian G. Cowell, Elise M. Ling, Rebecca L. Swan, Matilda L.W. Brooks, Caroline A. Austin

**Affiliations:** Institute for Cell and Molecular Biosciences, Newcastle University, Newcastle upon Tyne, United Kingdom

## Abstract

2,6-Diaminopyridine-3,5-bis(thiocyanate) (PR-619) is a broad-spectrum deubiquitinating enzyme (DUB) inhibitor that has been employed in cell-based studies as a tool to investigate the role of ubiquitination in various cellular processes. Here, we demonstrate that in addition to its action as a DUB inhibitor, PR-619 is a potent DNA topoisomerase II (TOP2) poison, inducing both DNA topoisomerase II*α* (TOP2A) and DNA topoisomerase II*β* (TOP2B) covalent DNA complexes with similar efficiency to the archetypal TOP2 poison etoposide. However, in contrast to etoposide, which induces TOP2-DNA complexes with a pan-nuclear distribution, PR-619 treatment results in nucleolar concentration of TOP2A and TOP2B. Notably, neither the induction of TOP2-DNA covalent complexes nor their nucleolar concentration are due to TOP2 hyperubiquitination since both occur even under conditions of depleted ubiquitin. Like etoposide, since PR-619 affected TOP2 enzyme activity in in vitro enzyme assays as well as in live cells, we conclude that PR-619 interacts directly with TOP2A and TOP2B. The concentration at which PR-619 exhibits robust cellular DUB inhibitor activity (5–20 *μ*M) is similar to the lowest concentration at which TOP2 poison activity was detected (above 20 *μ*M), which suggests that caution should be exercised when employing this DUB inhibitor in cell-based studies.

## Introduction

Ubiquitination is a post-translational modification where the polypeptide ubiquitin is attached to lysine residues of substrate proteins. This process is reversible, and ubiquitin removal is orchestrated by a large family of deubiquitinating enzymes (DUBs), which fall into six subfamilies based on sequence and domain similarity (Harrigan et al., 2018). Five of the subfamilies, which contain the majority of the DUBs, are cysteine peptidases. Ubiquitination and deubiquitination together with proteasomal activity control many aspects of cell physiology, and many of the enzymes involved have been implicated in human disorders, including cancer. As a result, they are an important focus of drug development programs (Huang and Dixit, 2016; Manasanch and Orlowski, 2017; Harrigan et al., 2018). A considerable number of small molecule inhibitors of specific DUBs are now in preclinical development (D’Arcy et al., 2015; Harrigan et al., 2018). These compounds are not only important as potential clinical leads but provide tools to probe the physiologic functions of specific DUBs and of ubiquitination in general. While most of the reported small molecule inhibitors exhibit specificity for particular DUBs, 2,6-diaminopyridine-3,5-bis(thiocyanate) (PR-619) is a broad-spectrum DUB inhibitor that nonetheless does not target other cysteine proteases such as cathepsin B or calpain 1 (Altun et al., 2011). In cell-based assays PR-619 was shown to substantially inhibit the activity of ubiquitin-specific protease, ubiquitin carboxy-terminal hydrolase, OTU, and MJD class DUBs when applied at 20–50 *µ*M, and exhibited a clear effect at a concentration as low as 5 *µ*M (Altun et al., 2011). PR-619 has been employed as a tool to investigate the role of ubiquitination in cellular processes including lysosomal degradation (Balut et al., 2011), caspase activation (Crowder et al., 2016), the stability of sirtuin-7 (Pandey and Kumar, 2015), protein aggregate formation (Seiberlich et al., 2012), human immunodeficiency virus replication (Setz et al., 2017), and oocyte maturation (Wang et al., 2017), as well as in the analysis of ubiquitin chain structure (Rana et al., 2017). In addition, PR-619 also inhibited the de-SUMOylating enzyme sentrin-specific protease (SENP) 6 in vitro and leads to accumulation of SUMOylated proteins in cells (Altun et al., 2011; Barry et al., 2018).

As described previously, PR-619 was characterized as a broad-spectrum DUB inhibitor (Altun et al., 2011). We report here that in addition to this activity, PR-619 affects DNA topoisomerase II (TOP2) and causes the accumulation of abundant DNA topoisomerase II*α* (TOP2A) and DNA topoisomerase II*β* (TOP2B) covalent DNA complexes in cells, with similar efficiency to the classic TOP2 poison etoposide. TOP2 enzymes alter DNA topology by forming a short-lived enzyme-bridged DNA double-strand break (DSB), where subunits of the dimeric TOP2 enzyme remain covalently attached to each end of the DSB via a 5′-phosphotyrosyl linkage. A second DNA segment then passes through the enzyme-bridged DNA gate, and finally the break is religated by the enzyme, completing the reaction cycle. TOP2 poisons, such as etoposide, are used in anticancer therapies; they inhibit the religation step of the enzyme’s reaction cycle, resulting in the persistence of covalently linked TOP2-DNA complexes (Cowell and Austin, 2012), which can be converted to DNA DSBs and are cytotoxic. These covalent complexes can be detected and quantified using the trapped in agarose DNA immunostaining (TARDIS) assay (Willmore et al., 1998; Cowell et al., 2011b; Cowell and Austin, 2018). We demonstrate here that PR-619 induces TOP2A and TOP2B covalent DNA complexes and redistribution of TOP2 in the nucleus. Surprisingly, we found that these effects occurred even under conditions of depleted ubiquitin, leading us to conclude that they are independent of the DUB inhibitory activity of PR-619, and thus probably result from direct interaction with TOP2, interfering with its religation activity.

## Materials and Methods

### 

#### Reagents and Antibodies.

Etoposide and 2′,3′,4′-trihydroxy-flavone (2-D08) were purchased from Sigma-Aldrich (Dorset, UK); PR-619 was obtained from Tocris Biosciences (Bristol UK); {(1*R*,2*R*,3*S*,4*R*)-2,3-dihydroxy-4-[(2-{3-[(trifluoromethyl)sulfanyl]phenyl}pyrazolo[1,5-a]pyrimidin-7-yl)amino]cyclopentyl}methyl sulfamate (MLN7243) was obtained from Active Biochem Ltd. (Hong Kong); and {(1*R*,2*S*,4*R*)-4-[(5-{[1-(3-bromobenzyl)-1*H*-pyrazol-3-yl]carbonyl}-4-pyrimidinyl)amino]-2-hydroxycyclopentyl}methyl sulfamate (ML-792) was obtained from Bioquote (York, UK). Rabbit anti-TOP2A (4566) and anti-TOP2B (4555) antibodies were raised in-house to the C-terminal domains of the respective proteins (Atwal et al., 2019). Anti-ubiquitin FK2 (anti-mono and poly-ubiquitinated conjugates, K^29^, K^48^, or K^63^ linked) was obtained from Enzo (Exeter, UK), and anti-SUMO2/3 (Ab81371) was from Abcam (Cambridge UK).

#### Cell Culture.

K562 cells were maintained in RPMI 1640 medium supplemented with 10% fetal bovine serum and 1% penicillin and streptomycin (Thermo Fisher Scientific, UK). HeLa cells were cultured in Eagle’s minimum essential medium plus 10% fetal bovine serum and 1% penicillin and streptomycin (Thermo Fisher Scientific). K562 (ATCC CCL-243) and HeLa (ECACC 93021013) cell lines were originally sourced from ATCC and ECACC, respectively. Cells were cultured at 37°C in a humidified atmosphere containing 5% CO_2_. Experiments were conducted on cells growing in log phase. Cells were routinely checked for mycoplasma infection.

#### Trapped in Agarose DNA Immunostaining Assay.

Cells were exposed for 2 hours to etoposide, PR-619, or an equal volume of DMSO and were then pelleted and washed in ice-cold PBS. For TARDIS assays involving HeLa cells, the cells were first trypsinized and briefly washed in cold complete cell culture medium. TARDIS assays were carried out essentially as described previously (Willmore et al., 1998; Cowell and Austin, 2018). TOP2 covalent complexes were visualized by immunofluorescence using rabbit anti-TOP2A (4566) or rabbit anti-TOP2B (4555) antibodies raised to the C-terminal domain of human TOP2A and TOP2B, respectively, and specific mouse antibodies as indicated. Secondary antibodies were AlexaFluor 488- or 594-coupled anti-rabbit or anti-mouse antibodies (Thermo Fisher Scientific). Slides were counterstained with Hoechst 33258 to visualize DNA. Hoechst and AlexaFluor images were captured using an epifluorescence microscope (Olympus IX-81) fitted with an Orca-AG camera (Hamamatsu) and suitable narrow band filter sets. For quantitative analysis, images were captured using a 10X objective. After image capture, automated slide scoring was performed using Volocity 6.3 software (PerkinElmer Inc., San Diego, CA) as described previously (Atwal et al., 2019) using the same parameters for each slide. Data were subsequently processed and represented using GraphPad Prism 8.2 (Perkin Elmer) and R. For higher resolution qualitative spatial analysis, a 40× or 60× objective was used and no blinding was applied. Image composites were made with Volocity 6.3 and Adobe Photoshop, ensuring that image adjustments were constant between different cell treatments.

#### Immunofluorescence Analysis Using Paraformaldehyde Fixed Samples.

K562 cells were washed and pelleted in ice-cold PBS and spotted onto poly-L-lysine reaction well slides (Marienfeld; VWR, Leicestershire, England). Cells were fixed in 4% formaldehyde in PBS at room temperature and permeabilized using 120 mM KCl, 20 mM NaCl, 10 mM Tris-HCl pH 8.0, 1 mM EDTA, and 0.1% Triton X-100 buffer. After blocking in 120 mM KCl, 20 mM NaCl, 10 mM Tris-HCl pH 8.0, 1 mM EDTA, and 0.1% Triton X-100 (plus 2% bovine serum albumin and 10% dry milk powder) buffer, cells were probed with primary and secondary antibodies as described for TARDIS. Slides were counterstained with 4,6-diamidino-2-phenylindole (Vector Laboratories, Burlingame, CA) and viewed using an epifluorescence microscope (Olympus IX-81). Atomated analysis and data presentation were performed as for TARDIS.

#### Differential Retention of TOP2 Analysis.

Differential retention of TOP2 (DRT) analysis was performed as described previously (Agostinho *et al.*, 2004; Cowell *et al.*, 2011a). Briefly, HeLa cells were grown on coverslips and then extracted once on ice in DRT buffer [30 mM HEPES, 65 mM PIPES, 10 mM EGTA, and 2 mM MgCl_2_ (pH 6.9), with 350 mM NaCl and 0.5% Triton X-100] containing protease inhibitors for 30 seconds to 1 minute with occasional gentle agitation. Cells were then fixed with paraformaldehye and imaged as described for immunofluorescence.

#### In Vitro TOP2 Assays.

Plasmid DNA cleavage reactions were performed as described previously (Burden et al., 2001). Each reaction contained 600 ng of purified TOP2A or TOP2B protein and 6.5 *μ*g plasmid DNA TCS1 (Lee et al., 1989). Precipitated DNA was resuspended in 15 *μ*l of water and 5 *μ*l agarose loading buffer (0.5% SDS, 25% glycerol, and 0.1% bromophenol blue). Samples were heated at 70°C for 2 minutes before loading onto a 1% Tris-acetate-EDTA agarose gel and electrophoresed at 45 V in 1× Tris-acetate-EDTA. After running, gels were stained with ethidium bromide and imaged under UV transillumination with Bio-Rad Gel Doc EZ Imager. For plasmid relaxation assays, 10 ng of purified TOP2A or TOP2B protein was incubated at 37°C for 30 minutes with 1.3 *μ*g supercoiled plasmid TCS1 in relaxation buffer [50 mM Tris (pH 7.5), 10 mM MgCl_2_, 0.5 mM EDTA, 30 *μ*g/ml bovine serum albumin, and 1 mM dithiothreitol) containing 1 mM ATP. Reactions were analyzed by electrophoresis in 0.8% Tris-borate-EDTA agarose gel. Gels were stained and imaged as previously described.

#### Chromatin Immunoprecipitation.

K562 cells were treated with 80 *µ*M PR-619, 100 *µ*M etoposide, or DMSO (control) for 2 hours, before collecting cells by centrifugation and washing twice with ice-cold PBS. Crosslinking was performed with 1% paraformaldehyde for 5 minutes on ice and paraformaldehyde was quenched with glycine. Cell pellets were frozen at −80°C. Pellets were thawed in 5 mM PIPES (pH 8.0), 85 mM KCl, and 0.5% NP40 plus protease inhibitors, and passed 10 times through a 21G syringe needle. Nuclei were pelleted and resuspended in 1% NP40, 0.5% Na deoxycholate, and 0.1% SDS in PBS containing protease inhibitors, and chromatin was sonicated to an average size of approximately 500 base pairs. Chromatin immunoprecipitation (ChIP) assays were performed with rabbit anti-TOP2A 4566, rabbit anti-TOP2B 4555, or control rabbit IgG prebound to protein A Dynabeads (Thermo Fisher Scientific). For semiquantitative polymerase chain reaction (PCR) analysis, cycle numbers were chosen that resulted in near endpoint yield for inputs (diluted 30× compared with immunoprecipitation samples) but clearly sub-endpoint yield for the test samples based on product band intensity and remaining primer visible as a lower band on the agarose gels. PCR products were resolved on 2% agarose gels stained with GelRed. For real-time quantitative PCR, reactions were performed using Ciba green and a Biorad CFX96 PCR machine using the ΔΔCt method. ChIP PCR primers have been described previously and were as follows: R-Prom f-GAGGACAGCGTGTCAGCAATAA, r-GCCCCGGGGGAGGTAT; FY-42 f-CTTTCCGGAGCTCTGCCTAG, r-GGTTGTCGGGCTCCATCT; OS-H4 f-CTCTCCGGAATCGAACCCTGA, r-CGACGACCCATTCGAACGTCT; OS-H8 f-CCCTTACGGTACTTGTTGACT, r-AGTCGGGTTGCTTGGGAATGC; OS-H18 f-GGAAGTTGTCTTCACGCCTGA, r-GTTGACGTACAGGGTGGACTG; Sa-GB f-AAGGTCAATGGCAGAAAAGAA, r-CAACGAAGGCCACAAGATGTC; Sa-GB f-AAGGTCAATGGCAGAAAAGAA, r-CAACGAAGGCCACAAGATGTC (O’Sullivan et al., 2002; Ray et al., 2013; Yu et al., 2015).

#### Data Analysis.

Statistical analysis was performed using GraphPad Prism 8.2. The details of the tests performed are given in the figure legends. For signifying *P* values, * refers to *P* < 0.05, ** refers to *P* < 0.01, *** refers to *P*< 0.001, and **** refers to *P*<0.0001. Error bars in the bar charts represent the S.D. values. The study was designed to be exploratory rather than testing a specific null hypothesis; therefore, the *P* values are descriptive only. Sample sizes (numbers of replicate experiments) were specified in advance of data acquisition based on prior knowledge of the characteristics of the assays involved and anticipating occasional lost or failed samples.

## Results

### 

#### PR-619 Induces TOP2A and TOP2B Covalent DNA Complexes.

**Fig. 1.**
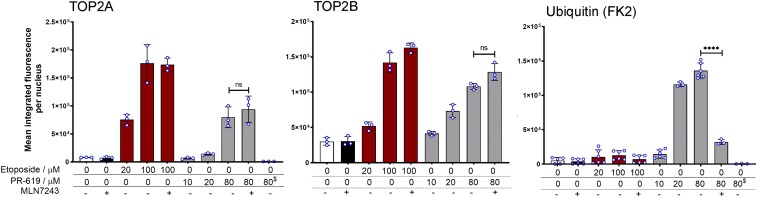
PR-619 induces TOP2 complexes in K562 cells with similar efficiency as etoposide, and complex formation is not dependent on ubiquitination. Cells were treated with PR-619, etoposide, or solvent control. Where indicated, cells were also treated with the E1 UEA inhibitor MNL7243 (10 *μ*M) prior to adding etoposide or PR-619. Cells were embedded in agarose on microscope slides and processed by TARDIS analysis for TOP2A, TOP2B, and ubiquitin. Bar graphs represent the mean of the median values derived from replicates, which are also indicated individually as blue-lined circles. Error bars represent the S.D. MNL7243 pretreatment was for 2 hours. Statistical analysis was performed using *t* tests. For the last column in the left and right panel highlighted (^$^ for the PR-619 concentration), cells were treated with 80 *μ*M PR-619 and processed with other samples, but with the omission of the primary antibody during immunofluorescence, to test for autofluorescence originating in the sample.

**Fig. 2.**
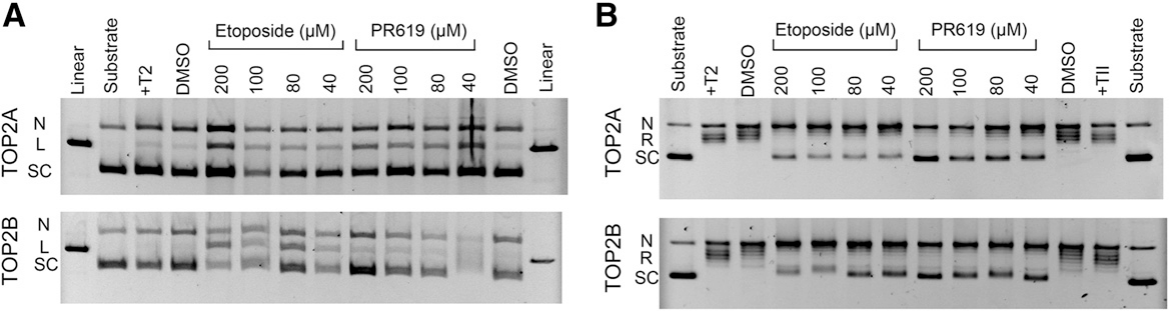
PR-619 inhibits TOP2-induced DNA cleavage and relaxation of supercoiling in vitro. (A) Etoposide and PR-619 induce plasmid cleavage by TOP2A or TOP2B. Plasmid cleavage was carried out with 600 ng recombinant TOP2A (top) or 600 ng of recombinant TOP2B (bottom). Positions of nicked (N), linear (L), and supercoiled plasmid (SC) are denoted on the left. Linearized plasmid cleaved with EcoR1 is shown in lane 1. Substrate only and substrate with TOP2 (+T2) are shown in lanes 2 and 3, respectively. As a control, reactions with 1 *μ*l DMSO (lane 4) and 2 *μ*l DMSO (lane 13) are shown. (B) Etoposide and PR-619 inhibited plasmid relaxation by TOP2A and TOP2B. Plasmid relaxation was carried out with 10 ng TOP2A (top) or 10 ng TOP2B (bottom). The positions of nicked (N), relaxed (R), and supercoiled plasmid (SC) are denoted on the left. Substrate only and substrate with TOP2 (+T2) are shown in lanes 1 and 2, respectively. As a control, reactions with 1 *μ*l DMSO (lane 3) and 2 *μ*l DMSO (lane 12) are included.

TOP2-DNA covalent complexes stabilized by drugs such as etoposide can be visualized and quantified using the TARDIS assay, which allows immunofluorescent analysis after removing cellular proteins, including histones, by high-stringency extraction of cells embedded in agarose, leaving nuclear ghosts of genomic DNA in situ (Supplemental Fig. 1, A–C). We had observed that etoposide-induced TOP2 DNA covalent complexes that are detected using this assay are accompanied by ubiquitin and SUMO immunofluorescence signals (Supplemental Fig. 1, B and C). When carrying out experiments to examine the ubiquitination of TOP2 in covalent DNA complexes, we noticed that the broad-spectrum DUB inhibitor PR-619 (Altun et al., 2011) itself induced both TOP2A- and TOP2B-DNA covalent complexes (Fig. 1; Supplemental Fig. 2, A and B, bottom six panels). As is the case for etoposide, PR-619 at 40 *µ*M induced an above-background TOP2A and TOP2B signal in nearly all cells (Supplemental Fig. 2, A and B). For TOP2A, the median signal intensity induced by 80 *µ*M PR-619 was approximately one-half that obtained with 100 *µ*M etoposide, and for TOP2B the intensity was 75% (Fig. 1). In contrast, at 10 *µ*M PR-619 did not induce detectable TOP2A complexes (Fig. 1; Supplemental Fig. 2C). Under standard TARDIS conditions TOP2A complexes are almost undetectable in non-drug-treated cells, whereas TOP2B reproducibly gives a small above zero signal in untreated cells; presumably reflecting the capture of endogenous TOP2B activity. Although PR-619 induced a clear induction of TOP2B complexes at 20, 40, and 80 *µ*M, little (if any) increase in TOP2B signal above that observed in untreated cells was observed in cells treated with 10 *µ*M PR-619 (Fig. 1, middle panel; Supplemental Fig. 1C). As described previously, it is possible to analyze TOP2 post-translational modifications using the TARDIS assay since the cell lysis and slide processing removes cellular constituents not covalently attached to genomic DNA. Using pan-ubiquitin antibodies, this revealed that at least some TOP2-DNA complexes induced by etoposide are ubiquitinated (Supplemental Figs. 1B and 2). Cells treated with 20, 40, or 80 *µ*M PR-619 exhibited a much higher ubiquitin signal than those treated with etoposide (Fig. 2, right panel; Supplemental Fig. 2), consistent with the activity of PR-619 as a DUB inhibitor. This leads to the conclusion that for complexes induced by PR-619 a higher proportion of the TOP2 molecules are ubiquitinated or that each TOP2 complex is ubiquitinated to a greater extent than those induced by etoposide. Notably, even at 10 *µ*M PR-619 some cells exhibited a high ubiquitin signal, although most nuclei remained at the untreated level (Supplemental Fig. 1C, right panel). This is surprising given that at this concentration PR-619 induced little (if any) additional TOP2A or TOP2B fluorescence above that observed in untreated cells but could be explained if 10 *µ*M PR-619 treatment results in hyperubiquitination of endogenous (i.e., nondrug-induced) TOP2B complexes. Thus, PR-619 behaves as an effective TOP2 poison, at a similar dose range as etoposide.

#### Induction of TOP2 DNA Covalent Complexes by PR-619 Is Not due to Hyperubiquitination of TOP2.

Since PR-619 is a broad range DUB inhibitor and treatment with PR-619 resulted in bright labeling of nuclear ghosts with anti-ubiquitin as well at TOP2 antibodies (Fig. 1, right panels; Supplemental Fig. 1, B and C), we hypothesized that the induction of TOP2-DNA complexes by PR-619 could be linked to its DUB inhibitory activity. To test this hypothesis, we pretreated cells with the E1 ubiquitin-activating enzyme inhibitor MLN7243 at a dose and time previously shown to cause a loss of polyubiquitin chains and monoubiquitinated histone H2A in cells (Hyer et al., 2018). As expected, this pretreatment greatly reduced the accumulation of ubiquitin in nuclear ghosts formed from PR-619–treated cells (Fig. 1, right panel). However, contrary to expectations, ubiquitin depletion by MLN7243 had no significant effect on PR-619–induced TOP2A or TOP2B complexes. MLN7243 did not induce TOP2-DNA complexes on its own, and also had no effect on etoposide-induced TOP2-DNA complex formation (Fig. 1, left and middle panels). Thus, PR-619 acts as a TOP2 poison independently of the effects of DUB inhibition. This conclusion is supported by in vitro enzyme assays, where like etoposide PR-619 stimulated TOP2A and TOP2B plasmid cleavage activity and inhibited decatenation activity (Fig. 2).

Etoposide treatment results in SUMO2/3 accumulation that coincides with TOP2 signal in the TARDIS assay (Schellenberg et al., 2017; Lee et al., 2018) (Supplemental Fig. 1C). Since PR-619 has been shown to inhibit SENPs (Altun et al., 2011), we were interested in determining whether PR-619 induced a proportionally greater level of SUMOylated protein-DNA covalent complexes. As shown in Supplemental Fig. 3, A–C, PR-619 did induce a robust SUMO2/3 signal, which coincided with the nuclear signal intensity observed for TOP2A and TOP2B, consistent with the presence of SUMOylated TOP2 complexes. However, we were not able to efficiently suppress the level of SUMOylation induced by PR-619 using small molecule inhibitors 2-D08 or ML-792, which affect the transfer of SUMO from E2 enzyme UBC9 or SAE1 activity, respectively (Kim et al., 2013; He et al., 2015).

#### PR-619 Induced TOP2A and TOP2B Covalent Complexes that Are Unevenly Distributed in the Nucleus.

**Fig. 3.**
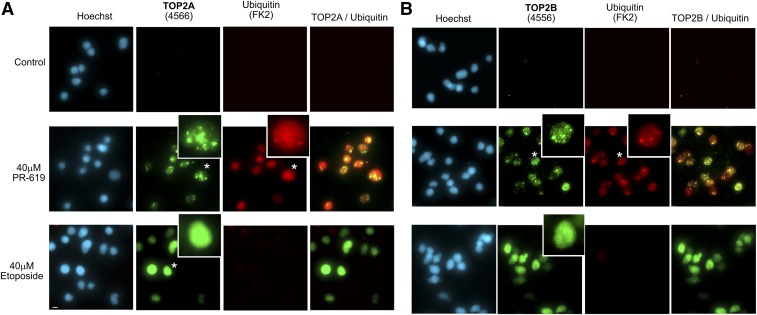
PR-619–induced TOP2 complexes are unevenly distributed in the nucleus. Cells were treated with PR-619, etoposide, or solvent control (DMSO) for 2 hours and TOP2 complexes were detected using the TARDIS assay. Immunofluorescence was performed using anti-TOP2A (A) or anti TOP2B (B) antibodies and anti-ubiquitin (FK2). Images were acquired using a 40X objective and are extended focus widefield images. Enlarged nuclei indicated by asterisk; scale bar, 10 *μ*m.

We routinely observe that etoposide and other established TOP2 poisons such as mitoxantrone induce TOP2A and TOP2B fluorescence signal throughout the volume of the nuclear ghosts in the TARDIS assay, although this can appear slightly granular for TOP2B under higher magnification widefield microscopy (Fig. 3). However, in most cells PR-619 induced a different distribution of TOP2A and TOP2B fluorescent signal consisting of large foci of signal (Fig. 3). The FK2 ubiquitin fluorescent signal partially overlapped that of both TOP2A and TOP2B (Fig. 3), as did that for SUMO2/3 (Supplemental Fig. 3A), consistent with the conclusion that the bright ubiquitin and SUMO signals originate from ubiquitinated and SUMOylated TOP2 trapped as covalent-DNA complexes.

#### PR-619 Drives Ubiquitin-Independent Nucleolar Localization of TOP2A and TOP2B.

**Fig. 4.**
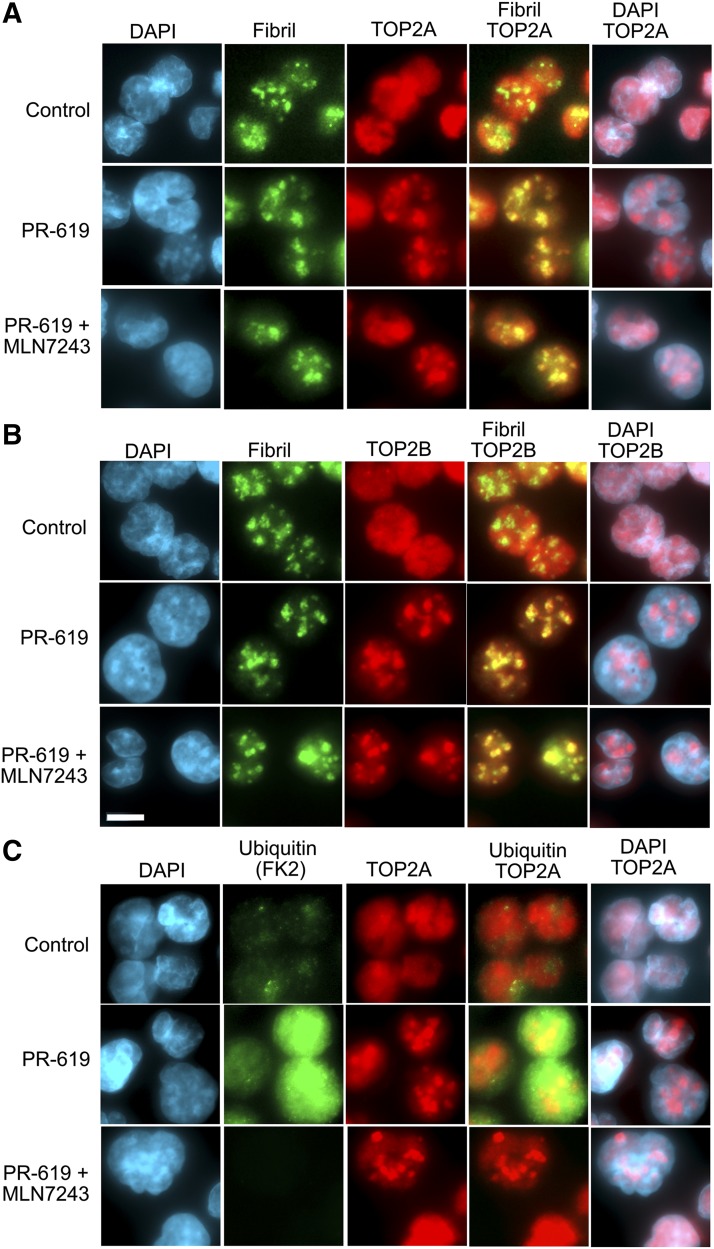
PR-619 drives nucleolar localization of TOP2A and TOP2B. K562 cells were treated with PR-619 (80 *µ*M), and where indicated were pretreated with the ubiquitin-activating enzyme inhibitor MLN7243 (10 *μ*M, 2 hours), fixed with paraformaldehyde, and analyzed by immunofluorescence for TOP2A (A) or TOP2B (B) and the nucleolar marker fibrillarin. (C) K562 cells were treated as in (A and B) and stained for ubiquitin (FK2) and TOP2A. Images are shown as extended focus projections using 0.1 *μ*m z-steps and representative images obtained from several fields of cells from duplicate slides; scale bar, 10 *μ*m.

Historically, there has been some disagreement regarding the subnuclear distribution of pools of TOP2, especially TOP2B, probably due to the use of cells from different species, antibodies, and fixation conditions (Chaly and Brown, 1996; Onoda et al., 2014; Austin et al., 2018). The consensus, in agreement with our own observations, is that TOP2 is mobile in the interphase nucleus and is distributed throughout the nucleoplasm with some distribution in the nucleoli. In addition, we have also observed a concentration of TOP2B in the abundant perinucleolar heterochromatin of mouse cells (Cowell et al., 2011a). Thus, we were interested to determine whether the uneven focal distribution of PR-619–induced TOP2A and TOP2B-DNA complexes in TARDIS assays reflected nucleolar concentration of TOP2. The harsh lysis conditions employed in the TARDIS assay remove proteinaceous nuclear structures, precluding costaining for nucleolar markers such as fibrillarin or pol I. Therefore, we examined the distribution of TOP2A and TOP2B in PR-619–treated K562 cells by standard immunofluorescence using paraformaldehyde fixation at room temperature. Under these fixation conditions both TOP2A and TOP2B display a fairly even nucleoplasm staining pattern in the majority of interphase cells. In contrast, in PR-619–treated cells TOP2A and TOP2B became concentrated in a few large nuclear foci, reminiscent of the pattern observed under TARDIS conditions (Figs. 3 and 4, A and B). Costaining for TOP2A or TOP2B and fibrillarin confirmed that the large focal TOP2 clusters in PR-619 colocalized with nucleoli (Fig. 4B). PR-619 induced a large increase in overall protein ubiquitination, as detected using antibody FK2 in paraformaldehyde fixed cells, but prior treatment with the ubiquitin-activating enzyme inhibitor MLN7243 resulted in almost complete loss of FK2 signal, even in PR-619–treated cells (Fig. 4C). However, the PR-619–associated nucleolar redistribution of TOP2 was not affected by prior treatment with MLN7423. Thus, the nucleolar redistribution of TOP2A and TOP2B in PR-619–treated cells does not appear to be dependent on hyperubiquitination of the enzymes.

Sites of TOP2 activity can be visualized using the DRT assay (Agostinho et al., 2004), in which adherent cells grown on coverslips are extracted with salt and detergent to enrich for active TOP2 molecules that are trapped on DNA. Since overall nuclear architecture is better conserved by DRT assay than in the TARDIS assay, this method was used to examine the nuclear distribution of active TOP2A and TOP2B in HeLa cells. In the absence of added drug, most TOP2A and TOP2B were lost from the cells, as expected (Supplemental Figs. 4 and 5, top rows). However, PR-619 treatment led to the formation of focal concentrations of both TOP2A and TOP2B corresponding to the location of nucleoli as judged from the 4,6-diamidino-2-phenylindole staining pattern. In contrast, but consistent with previous observations, etoposide induced an essentially pan-nuclear TOP2A pattern and a fine granular pattern with TOP2B. Thus, PR-619 traps TOP2A and TOP2B in the nucleolar compartment of the epithelial cells (HeLa) as well as lymphoblastoid cells (K562). To be trapped and stable to extraction, TOP2 must presumably be active in the nucleolar compartment of PR-619–treated cells. Notably, Onoda et al. (2014) demonstrated that a pool of TOP2B is localized in the nucleolar regions in live cells and after fixing with formaldehyde at 37°C, but redistributes to the nucleoplasm if cells are cooled; these authors report that this nucleolar pool is largely inactive and bound to RNA. To determine whether TOP2 is concentrated in the nucleolar domain via RNA interactions in PR-619–treated cells, K562 cells were treated with PR-619 or etoposide and TOP2 complexes were visualized and quantified using the TARDIS assay after treating extracted agarose-embedded cells with RNase A. Notably, RNase treatment made no difference to either the qualitative distribution or the intensity of the TOP2A or TOP2B signals (Supplemental Fig. 6).

**Fig. 5.**
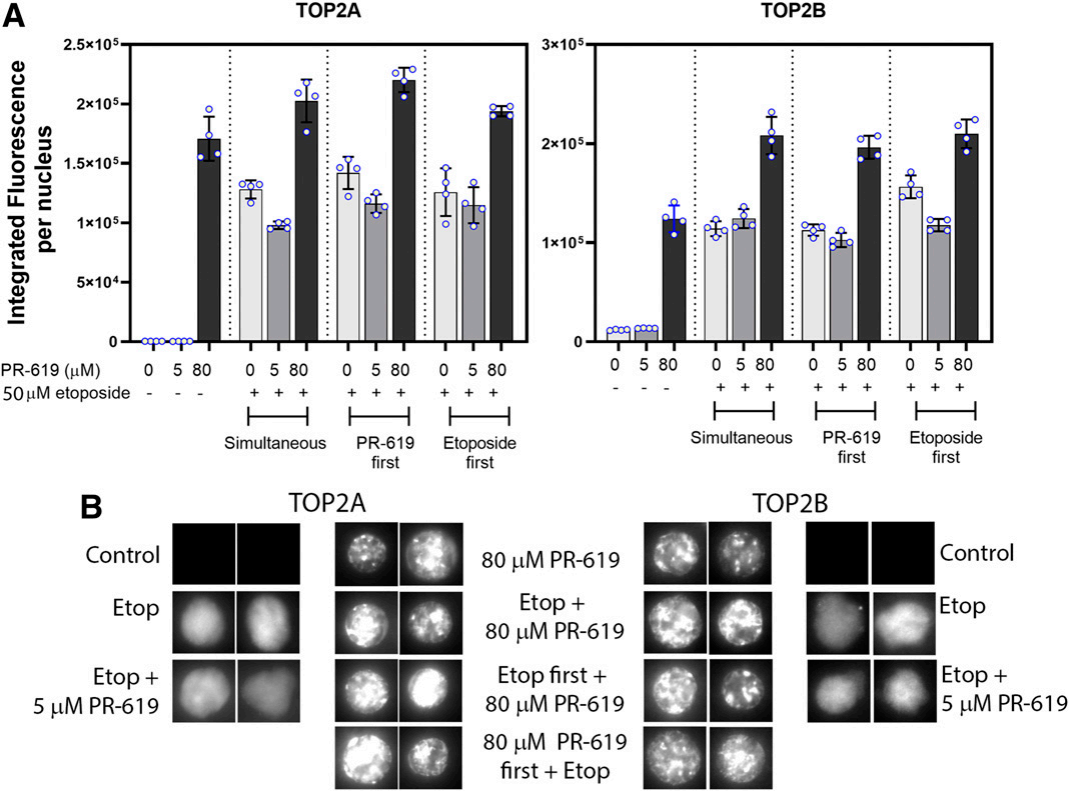
Combined etoposide and PR-619 treatment. K562 cells were treated with etoposide or PR-619 individually for 150 minutes, with both for 150 minutes or with either one of the drugs first for 30 minutes before adding the other drug for 120 minutes and then collecting the cells for TARDIS analysis. (A) Quantitative analysis of TARDIS samples as described for Fig. 1. (B) Representative nuclei from extended focus images acquired at higher magnification.

Since etoposide and PR-619 induce TOP2-DNA complexes with different distributions in the nucleus, we carried out combination experiments to determine whether complex formation would be quantitatively (in the overall level of complexes formed) or qualitatively (in the distribution of TOP2 complexes in the nucleus) additive. Using both drugs at concentrations that individually generate similar levels of TOP2 complexes (50 *µ*M etoposide and 80 *µ*M PR-619), we found that for TOP2B the combination of both drugs resulted in approximately 30% larger signal than either drug alone, whether the drugs were added simultaneously or sequentially (Fig. 5A). For TOP2A, the combination resulted in only a marginal increase in signal compared with PR-619 alone (Fig. 5A). Thus, there is some additive effect, particularly for TOP2B, but this may have been limited by saturating effects at the concentrations of drugs used. While etoposide and PR-619 treatment resulted in different distributions of TOP2A and TOP2B within nuclear ghosts (Fig. 5B) combined treatment with both drugs resulted in TOP2A and TOP2B complex distributions very similar to that obtained with PR-619 alone (Fig. 5B; Supplemental Figs. 7 and 8). In particular, pretreatment with etoposide did not noticeably prevent the focal distribution associated with PR-619, suggesting that a sufficient pool of untrapped TOP2 remains after 50 *µ*M etoposide treatment to subsequently give rise to the focal PR-619–derived pattern of TOP2 complexes. Also of note, pretreatment with 5 *µ*M PR-619, sufficient to inhibit DUB and SENP activity (Altun et al., 2011) but insufficient to generate TOP2-DNA complexes, did not affect the distribution of TOP2 complexes induced by etoposide.

#### TOP2A and TOP2B Associate with Recombinant DNA Repeats.

**Fig. 6.**
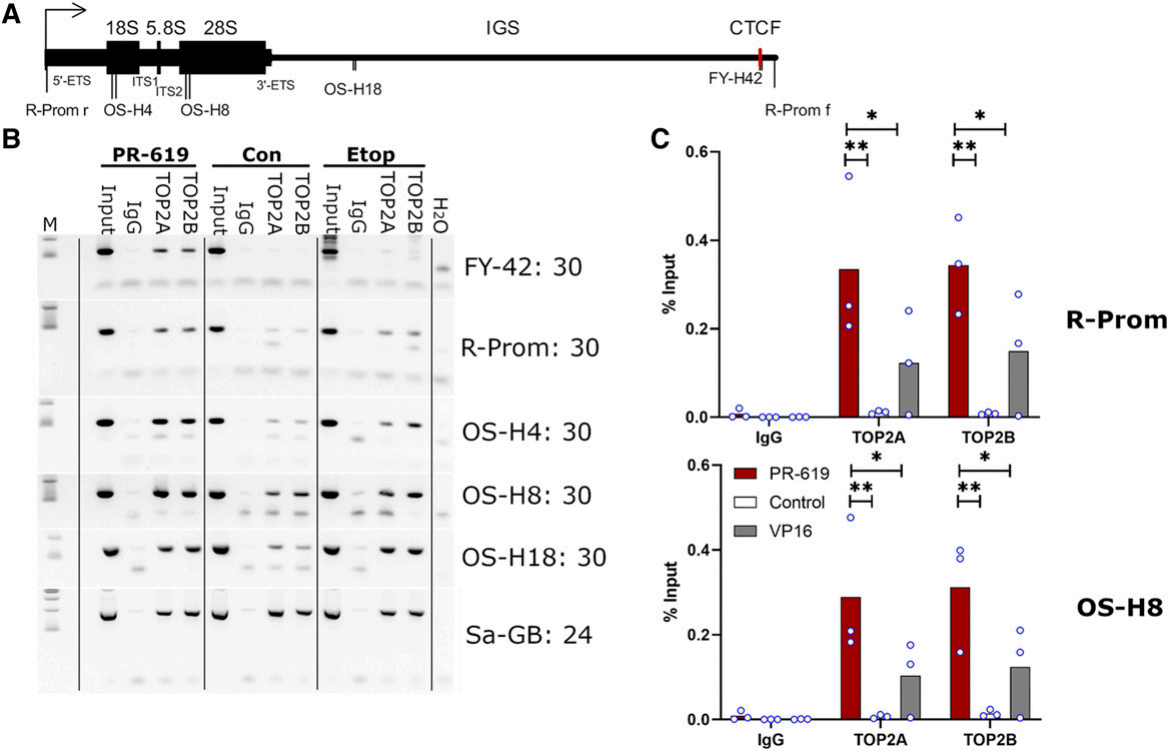
PR-619 treatment leads to enhanced association of TOP2A and TOP2B with rDNA. (A) Representation of the human rDNA repeat unit showing promoter (arrow), coding sequence (thick line), CTCF site (red bar), and PCR primers. (B) Semiquantitative PCR from ChIP DNA samples from untreated (control), PR-619–treated, and etoposide-treated K562 cells. Track M: 100 and 200 base pair markers; input = 1/30 dilution of input chromatin used for immunoprecipitation, IgG control, and rabbit IgG. Primer pair names and the number of PCR cycles are indicated on the right. (C) Quantitative real-time PRC analysis for primers R-prom (promoter) and OS-H8 coding region corresponding to 28S RNA.

Given that PR-619 treatment leads to nucleolar retention of TOP2 (Fig. 4) and induces TOP2-DNA complexes that appear to coincide with nucleolar-derived regions in TARDIS and DRT assays, we set out to determine whether PR-619 drives TOP2 association with ribosomal DNA (rDNA) repeats. Notably, previous studies have demonstrated that TOP2B is present across at least some rDNA repeat units by ChIP sequencing (Uusküla-Reimand et al., 2016) and that TOP2A associates with and promotes the activity of pol I at rDNA promoters (Ray et al., 2013). We performed ChIP analysis with brief formaldehyde crosslinking, utilizing PCR primers corresponding to the rDNA promoter, 18S, and 28S coding regions and the spacer region (Fig. 6A). Under control conditions TOP2A and TOP2B could be detected at each of these locations using semiquantitative PCR, but the signal was more robust with either TOP2 poison, particularly with PR-619 (Fig. 6B). This was confirmed by quantitative PCR for two of the locations (promoter and 28S) (Fig. 6C). Thus, PR-619 does appear to lead to increased association of TOP2A and TOP2B with rDNA. For comparison, we examined the association of TOP2 with satellite DNA (some of which occupies a perinucleolar position in the nucleus) using an alpha satellite PCR primer pair. We observed a robust TOP2A and TOP2B ChIP signal under each condition but did not observe additional PR-619–mediated accumulation for alpha satellite DNA (Fig. 6B).

#### PR-619 Efficiently Induces Histone H2AX Phosphorylation.

**Fig. 7.**
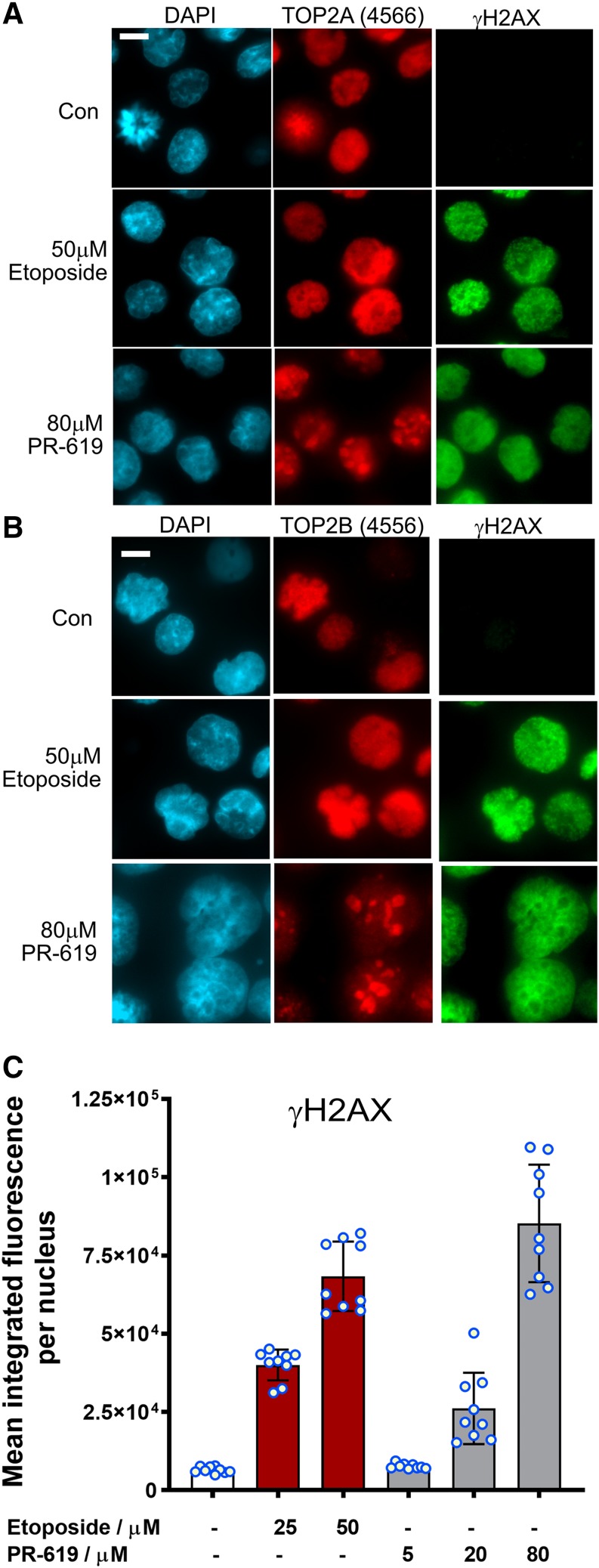
PR-619 induces histone H2AX phosphorylation. (A and B) K562 cells were treated with etoposide (50 *µ*M) or PR-619 (80 *µ*M) for 2 hours, fixed with paraformaldehyde, and TOP2A or TOP2B and *γ*H2AX were detected by immunofluorescences. Images are representative examples from three replica experiments. (C) H2AX phosphorylation per nucleus was assessed by quantitative immunofluorescent. Data represent mean ± S.D., individual data points are indicated as blue-lined circles; scale bar, 10 *μ*m.

Cellular processing of TOP2-DNA complexes via proteasomal destruction and other mechanisms leads to the appearance of protein-free DSBs, which are otherwise concealed by TOP2 protein and do not elicit a DNA damage response (Mårtensson et al., 2003; Zhang et al., 2006; Fan et al., 2008; Schellenberg et al., 2017; Lee et al., 2018). As a proxy for DSBs, we measured the appearance of phospho-histone H2AX (*γ*H2AX) in K562 cells treated with etoposide or PR-619. Both etoposide and PR-619 induced robust H2AX phosphorylation at doses that efficiently trap TOP2 in covalent-DNA complexes (Fig. 7; Supplemental Fig. 9). However, comparing equal doses of PR-619 and etoposide, PR-619 exhibited a much steeper dose response than etoposide, producing little *γ*H2AX in most cells at 20 *µ*M and background levels at 5 and 10 *µ*M.

#### PR-619 Induces Persistent TOP2-DNA Complexes.

**Fig. 8.**
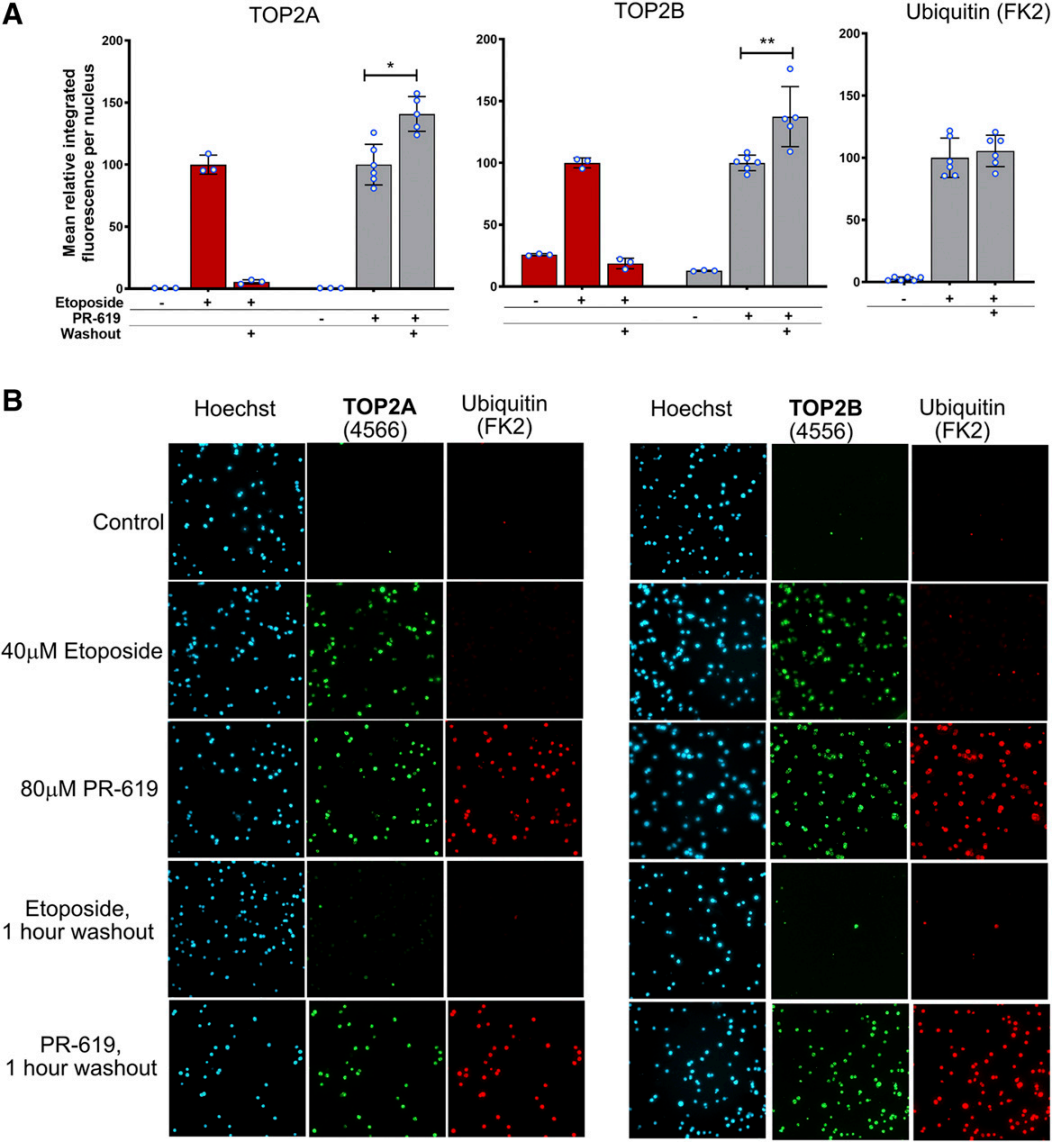
PR-619–induced TOP2 complexes persist after drug washout. K562 cells were incubated in the presence of etoposide (40 *µ*M), PR-619 (80 *µ*M), or solvent (DMSO) for 2 hours. Cells were either collected immediately or drug was washed out and cells were replated and incubated for a further 1 hour before collection for TARDIS analysis. (A) Quantification of TARDIS data obtained using anti-TOP2A (4566), anti-TOP2B (4556), and anti-ubiquitin (FK2) antibodies. Median-integrated fluorescence per nucleus values were normalized to the mean of the medians obtained with each drug directly after 2-hour incubation (i.e., without washout). Data are shown as mean ± S.D. values, and values from individual replicas are indicated as blue-lined circles. Statistical analysis, the normalized mean integrated fluorescence for PR-619 before and after washout were compared by unpaired *t* test. (B) Representative images from TARDIS slides used to produce part A.

The resolution of poison-induced TOP2-DNA covalent complexes in cells after drug washout can be followed using TARDIS, immunocomplex of enzyme, or potassium-SDS precipitation assays (Hsiang and Liu, 1989; Errington et al., 2004; Nitiss et al., 2012; Schellenberg et al., 2017). In cultured mammalian cells, the half-life of etoposide-induced TOP2A and TOP2B complexes is less than an hour (Errington et al., 2004; Schellenberg et al., 2017). However, the observed reversal rate differs for different TOP2 poisons. While the mAMSA reversal rate is similar to etoposide, it is much longer for mitoxantrone (Fox and Smith, 1990; Willmore et al., 2002), and in the case of the anthracycline idarubicin, we observed TOP2-covalent DNA complexes increase for up to 48 hours after drug washout (Errington et al., 2004). To determine the longevity of PR-619–-induced TOP2 complexes, K562 cells were incubated with etoposide or PR-619 for 2 hours as before, and then collected for TARDIS analysis before and after drug washout (1 hour) and replated in fresh medium. In line with the rapid reversal of etoposide-induced TOP2 DNA complexes observed previously, most of the etoposide-induced TOP2A signal was lost and the TOP2B complexes returned to background levels following drug washout. In contrast, in K562 cells treated with PR-619, TOP2A and TOP2B signals increased significantly following drug washout, while the ubiquitin signal was maintained (Fig. 8). The reason for the persistence of the PR-619–induced complexes could include slower cellular processing of these complexes compared with those induced by etoposide; however, the appearance of abundant *γ*H2AX (similar to the signal obtained with 50 *µ*M etoposide) during the 2-hour drug incubation period (Fig. 7; Supplemental Fig. 9) indicates that a substantial fraction of PR-619–induced TOP2 complexes are processed to reveal protein-free breaks during this period. Alternatively, greater retention of PR-619 in K562 cells following drug washout could lead to persistence of TOP2-DNA complexes, an explanation that has been suggested for the persistence of mitoxantrone- and idarubicin-induced TOP2-DNA complexes (Willmore et al., 2002; Errington et al., 2004). In this scenario, significant PR-619 is retained in cells during drug washout, resulting in continued formation of new TOP2-DNA complexes even as the initial complexes are processed to DSBs. In support of this, the yellow color of PR-619 was clearly visible in cell pellets of PR-619–treated cells after washing with PBS.

## Discussion

We have demonstrated that PR-619, a previously characterized broad-spectrum DUB inhibitor (Altun et al., 2011), is also a TOP2 poison, inducing TOP2A and TOP2B DNA complexes with similar potency to the archetypal and clinically important TOP2 poison etoposide. Established TOP2 poisons fall into a number of chemical classes including podophyllotoxins such as etoposide and teniposide, the anthraciendiones mitoxantrone and pixantrone, anthracyclines such as idarubicin, acridines including mAMSA, and the quinolone Voreloxin (Pommier et al., 2010). However, PR-619 is chemically distinct from each of these classes of TOP2 poison. TOP2-DNA complexes induced by PR-619 were highly ubiquitinated. However, TOP2-DNA covalent complexes were formed in PR-619–treated cells even in the presence of the ubiquitin-activating enzyme inhibitor MLN7243, although the level of ubiquitination of the complexes was much lower in MLN7243 pretreated cells. Thus, it does not appear that hyperubiquitination of TOP2A or TOP2B is a prerequisite for the formation of TOP2-DNA complexes. In a previous study using HCT-116 cells (Hyer et al., 2018), and as demonstrated here in K562 cells (Fig 4C), MLN7243 caused a very large reduction in protein ubiquitination within 2 hours (the length of preincubation employed in this study). Therefore, it is unlikely—although it cannot be fully excluded—that hyperubiquitination of another protein interferes with normal TOP2 activity leading to TOP2-DNA complex formation. We found TOP2 complexes induced by etoposide are decorated with SUMO2/3 in addition to ubiquitin (Supplemental Figs. 1C and 3), as observed previously (Agostinho et al., 2008; Schellenberg et al., 2017; Lee et al., 2018); however, the intensity of SUMO staining was greater in PR-619–treated cells, consistent with the activity of the inhibitor against SENPs. Thus, it is plausible that PR-619 induces TOP2 complexes via hyper-SUMOylation of the enzymes or another cellular component. We were unable to fully exclude this possibility in cell-based studies since pretreatment with either 2-D08 or ML-792 did not substantially affect the SUMO2/3 signal induced in nuclear ghosts by PR-619 (Supplemental Fig. 3). However, since PR-619 induced DNA cleavage and inhibited relaxation activities of TOP2A and TOP2B in in vitro cleavage assays (Fig. 2) PR-619–induced formation of TOP2A and TOP2B-DNA complexes in cells is likely to be a direct action on TOP2 enzyme activity. In addition to inducing TOP2-DNA complexes, PR-619 uniquely caused a redistribution of TOP2 into the nucleolar compartment, observed by standard immunofluorescence employing paraformaldehyde fixation and in TARDIS imaging. This phenomenon occurred even when cells were pretreated with MLN7243, and thus also appears to be independent of the DUB inhibitor activity of PR-619. However, for the reasons described previously, we are not able to exclude the possibility that hyper-SUMOylation of TOP2 contributes to its nucleolar accumulation. Although, arguing against this, we observed that PR-619 at a concentration (5–10 *μ*M) that achieves efficient DUB and SENP inhibition in cells (Altun et al., 2011) did not affect the diffuse nuclear distribution of etoposide-induced TOP2-DNA complexes (Fig. 5). Notably, SUMOylation of TOP2 is associated with targeting to mitotic chromosomes (Azuma et al., 2005; Agostinho et al., 2008), and artificial fusion of poly-SUMO to yeast Top2 results in nucleolar targeting (Takahashi and Strunnikov, 2008). Although TOP2A and TOP2B are found throughout the interphase nucleus by immunofluorescence in fixed cells they are both components of the nucleolar proteome (Andersen et al., 2005). In addition, protein interactions between TOP2B and a number of nucleolar proteins have been demonstrated (Uusküla-Reimand et al., 2016) and live cell imaging revealed a dynamic and more nucleolar distribution of TOP2A and TOP2B GFP fusion proteins (Christensen et al., 2002; Onoda et al., 2014). Thus, it is reasonable to conclude that PR-619 influences the normal distribution of TOP2 between the nucleoplasm and nucleoli, although the mechanism behind this is currently unclear. Notably, to form complexes resistant to extraction in TARDIS and DRT assays TOP2A and TOP2B must be enzymatically active in the nucleolar compartment in PR-619–treated cells. TOP2A and TOP2B have been reported to associate with rDNA sequences, and in ChIP-sequencing analysis TOP2B occupancy was evident across the rDNA repeat unit coding region and at the promoter and adjacent CTCF binding region (Ray et al., 2013; Uusküla-Reimand et al., 2016). Although we could detect TOP2A and TOP2B at the promoter and coding regions of the rDNA repeat unit in untreated cells even with the relatively mild crosslinking conditions employed (Fig. 6B), ChIP efficiency was greater when TOP2 was trapped on DNA by etoposide, or PR-619, but this effect was more robust in the case of PR-619 (Fig. 6, B and C). This is consistent with PR-619 resulting in abundant TOP2-DNA complexes within rDNA repeat units, although our evidence suggests that these complexes are distributed across the locus.

PR-619 has become a useful tool in order to probe the role of ubiquitination in various cell biologic systems (Balut et al., 2011; Seiberlich et al., 2012; Pandey and Kumar, 2015; Crowder et al., 2016; Rana et al., 2017; Wang et al., 2017); however, the work here suggests that some caution should be applied when using this inhibitor in cell line–based studies. We have observed that at concentrations 20 *µ*M and above, PR-619 induces TOP2-covalent DNA complexes in K562 cells that are converted to DNA double-strand breaks. Notably, the concentration range where we have observed pronounced TOP2 poison activity and *γ*H2AX induction in K562 cells (20–80 *µ*M) is higher than the lowest concentrations for which PR-619 has demonstrated robust DUB inhibitory activity (5–20 *µ*M) and growth inhibitory activity (IC_50_ ∼2 *µ*M) in HEK 293T cells (Altun et al., 2011). Thus, the additional TOP2 poisoning property of PR-619 can be avoided by careful consideration of concentration.

## Abbreviations

ChIP: chromatin immunoprecipitation
DRT: differential retention of TOP2
DSB: double-strand break
DUB: deubiquitinating enzyme
MLN7243: {(1*R*,2*R*,3*S*,4*R*)-2,3-dihydroxy-4-[(2-{3-[(trifluoromethyl)sulfanyl]phenyl}pyrazolo[1,5-a]pyrimidin-7-yl)amino]cyclopentyl}methyl sulfamate
PCR: polymerase chain reaction
PR-619: 2,6-diaminopyridine-3,5-bis(thiocyanate)
rDNA: ribosomal DNA
SENP: sentrin-specific protease
TARDIS: trapped in agarose DNA immunostaining
TOP2: DNA topoisomerase II
TOP2A: DNA topoisomerase II*α*
TOP2B: DNA topoisomerase II*β*
